# Forces Engaged in the Circulation of the Blood

**Published:** 1888-10

**Authors:** C. W. Spalding

**Affiliations:** St. Louis


### FORCES ENGAGED IN THE CIRCULATION OF THE BLOOD.

C. W. Spalding, M. D., St. Louis.

In the *American Homœopathist*, Dr. A. M. Cushing asks: "How does the blood circulate?" and after having seen the blood circulate in the arteries of a fly's wing, after it was torn from the body, concludes that the muscles of arteries "can continue the circulation without the assistance of the heart."

That the muscles of all arteries *aid* in the circulation is a well-established fact in physiology, but there are other forces that also contribute to the same phenomenon. Besides the rhythmical contractions of the heart, which may be regarded as the central circulatory force; the respiratory movements of the lungs, voluntary and involuntary; the contraction of voluntary muscles and the reflex force of the capillaries may be mentioned. "An attraction exists between all the fluids of the body and their respective destination and uses, and in the case of the blood a manifest attraction inviting that fluid away from the parts that do not require it toward those that do." That there is a living power in the tissues which invites and attracts the arterial blood, and at the same time repels the exhausted venous blood, can scarcely be doubted.

In speaking of the circulation of the blood, Michael Foster, than whom we have no higher *acknowledged* authority, says: "The heart is a pump, the motive power of which is supplied by the contraction of its fibres. Regarded as a pump, its effects are determined by the frequency of its beats, by the force of each beat, and by the quantity of fluid ejected at each beat. The quantity ejected at each beat is governed more by the state of the rest of the body than by that of the heart itself." Excepting the latter clause, I think this view must be regarded as fallacious. The muscular force of the heart is, of itself, insufficient to maintain the circulation of the blood. In addition to the forces already mentioned, there is another, more important in its action than the combined action of all the above-named auxiliary forces. I allude to the life-force resident in the blood. That the life of the body is in the blood, is evident from the fact that any function of the body other than the circulation of the blood may be suspended for a considerable time and resuscitation is still possible. Respiration may cease and all our conscious life be suspended, but so long as the blood moves life remains.

In the ordinary process of dying the motions of the heart, especially the systolic, sometimes continue long after respiration ceases, and all other outward signs of life disappear. The diastole of the heart is first enfeebled and grows weaker and weaker until the blood flows through the auricles, almost without perceptible contraction of these chambers. The systolic motion is the last to expire, and so long as this continues, even at long intervals, circulation is possible, unless prevented by other unfavorable conditions.

In vegetables, sap circulates in the absence of special organs, and in the lower animals the blood circulates without the *existence* of a heart, and in cold-blooded animals possessed of a heart, the movement of the blood continues after its excision and removal, as was the case with the fly's wing after it was torn from the body.

The onflow of the blood in the venæ cavæ causes an expansion of the auricles in the heart's diastole, and the pressure of the blood coming from the auricles partly fills the ventricles before contraction seizes the base of the heart. This movement of the blood, which is strong enough to open and command the heart valves, should not be attributed to the contractile force of the heart. The source of power in such case would, by its remoteness, be too uncertain for so vital an organ.

Is it, then, too much to assume that a vital circulatory force dwells in the blood itself? Harvey, in speaking of the egg, says: "First of all there is in it a drop of blood which palpitates, and from which are formed the auricles of the heart." As to the auxiliary forces there is not a point or function in the circulatory system but contributes to the movement of the blood.

